# Next steps in the identification of gene targets for type 1 diabetes

**DOI:** 10.1007/s00125-020-05248-8

**Published:** 2020-08-14

**Authors:** Struan F. A. Grant, Andrew D. Wells, Stephen S. Rich

**Affiliations:** 1 Center for Spatial and Functional Genomics, The Children’s Hospital of Philadelphia, Philadelphia, PA USA; 2 Departments of Pediatrics and Genetics, University of Pennsylvania Perelman School of Medicine, Philadelphia, PA USA; 3 Divisions of Human Genetics and Endocrinology, The Children’s Hospital of Philadelphia, Philadelphia, PA USA; 4 Department of Pathology and Laboratory Medicine, University of Pennsylvania Perelman School of Medicine, Philadelphia, PA USA; 5 Center for Public Health Genomics, University of Virginia School of Medicine, Charlottesville, VA USA; 6 Department of Public Health Sciences, University of Virginia School of Medicine, Charlottesville, VA USA

**Keywords:** Chromatin, Enhancers, eQTLs, Genetics, Prediction, Review, Target genes, Type 1 Diabetes

## Abstract

The purpose of this review is to provide a view of the future of genomics and other omics approaches in defining the genetic contribution to all stages of risk of type 1 diabetes and the functional impact and clinical implementations of the associated variants. From the recognition nearly 50 years ago that genetics (in the form of HLA) distinguishes risk of type 1 diabetes from type 2 diabetes, advances in technology and sample acquisition through collaboration have identified over 60 loci harbouring SNPs associated with type 1 diabetes risk. Coupled with HLA region genes, these variants account for the majority of the genetic risk (~50% of the total risk); however, relatively few variants are located in coding regions of genes exerting a predicted protein change. The vast majority of genetic risk in type 1 diabetes appears to be attributed to regions of the genome involved in gene regulation, but the target effectors of those genetic variants are not readily identifiable. Although past genetic studies clearly implicated immune-relevant cell types involved in risk, the target organ (the beta cell) was left untouched. Through emergent technologies, using combinations of genetics, gene expression, epigenetics, chromosome conformation and gene editing, novel landscapes of how SNPs regulate genes have emerged. Furthermore, both the immune system and the beta cell and their biological pathways have been implicated in a context-specific manner. The use of variants from immune and beta cell studies distinguish type 1 diabetes from type 2 diabetes and, when they are combined in a genetic risk score, open new avenues for prediction and treatment.

**Electronic supplementary material:**

The online version of this article (10.1007/s00125-020-05248-8) contains a slide of the figure for download, which is available to authorised users.

**Figure:**
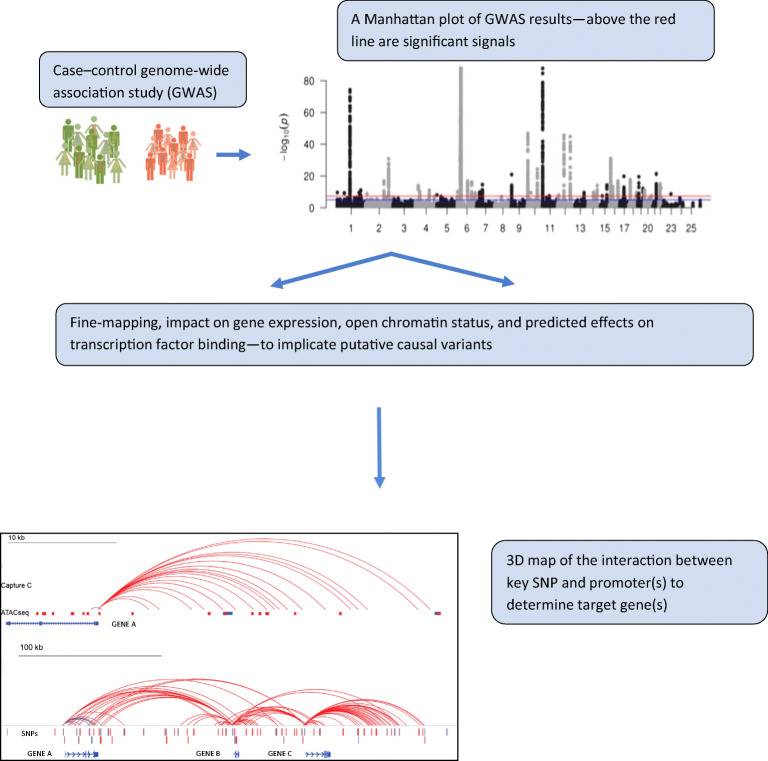
Graphical abstract

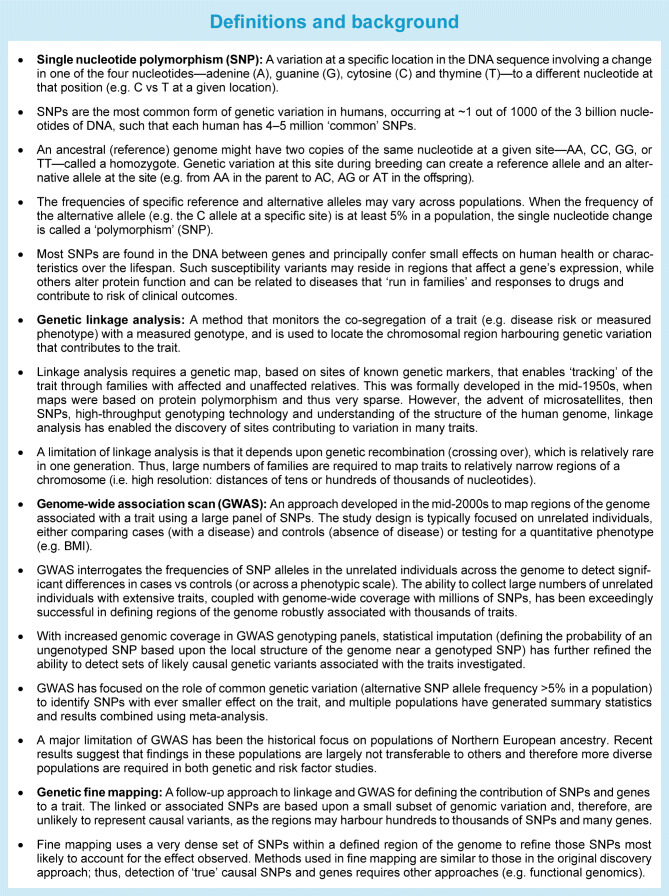

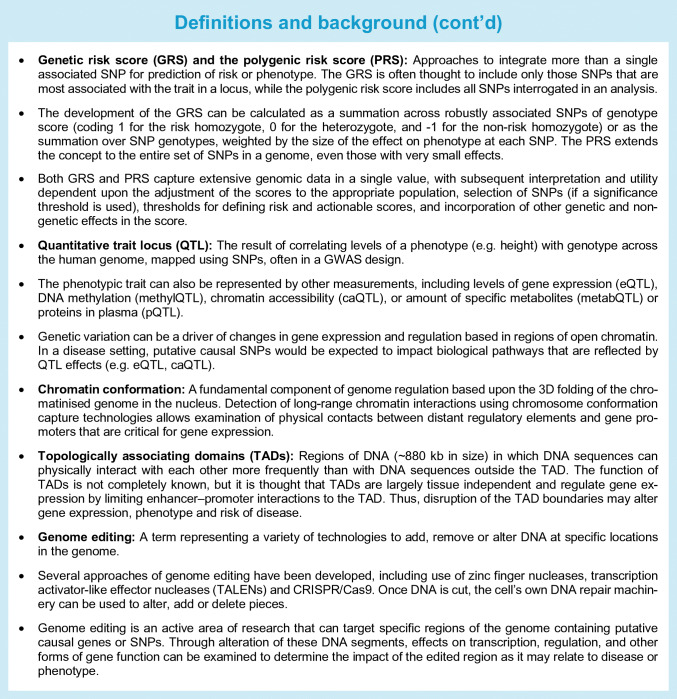

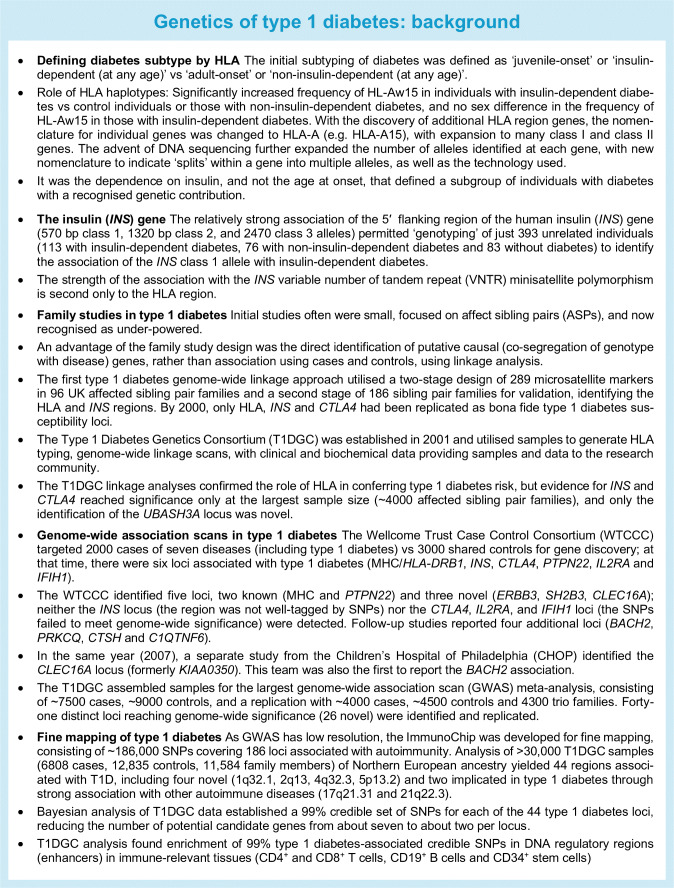







## Introduction

Diabetes is a clinically heterogeneous, chronic condition characterised by a failure to maintain normal glucose levels through conversion of food into energy via insulin-dependent mechanisms. The most common forms of diabetes have been defined by clinical differences in insulin dependence to maintain glucose homeostasis, the age and abruptness of onset of symptoms, and tendency for ketosis. This review will provide background on the genetic basis of type 1 diabetes, the function of genetic variation, and future work moving to discovery of target genes, pathways and mechanisms, novel interventions and the identification of therapeutic targets.

## Genetic basis of type 1 diabetes

Following the discovery of type 1 diabetes associated with HLA [1], the insulin (*INS*) variable number tandem repeat (VNTR) [2], and numerous candidate gene polymorphisms, the development of high-throughput genotyping array technology and analytical methods expanded our knowledge of genetic variation implicated in type 1 diabetes risk (summarised in the Text box ‘Genetics of type 1 diabetes: background’). Despite decades of interrogating HLA, much remains to be understood about specific allelic and interaction effects within that region [3] across populations. The Wellcome Trust Case Control Consortium (WTCCC) established the genome-wide association scan (GWAS) as a primary tool in discovery of genetic variants associated with common disease [4]; however, the WTCCC identified relatively few novel risk loci (*ERBB3*, *SH2B3*), including one simultaneous discovery (*CLEC16A*, formerly known as *KIAA0350*) [5]. Later, the Type 1 Diabetes Genetics Consortium (T1DGC) conducted the largest GWAS meta-analysis of type 1 diabetes in ~7500 cases and ~9000 controls, with replication in ~4000 cases, ~4500 controls and 4300 trio families [6]. The T1DGC identified 41 distinct loci, including 26 that were novel.

## Fine mapping type 1 diabetes-associated loci identified from GWAS

As with other GWAS, the T1DGC GWAS meta-analysis [6] yielded loci that were large (~250 kb for each locus), with many genes (ranging from 0 to 28) commonly harboured within each corresponding region [7]. To get as close to the underlying causal variants underlying these associations, fine mapping employing genotyping arrays with dense coverage within each locus (ImmunoChip) was performed on >30,000 individuals (cases, controls and families) [8]. Credible sets of SNPs were established for each of the 44 loci, revealing enrichment of SNPs in DNA regulatory regions. These results supported a role for enhancer chromatin states in immune-relevant cell types (CD4^+^ and CD8^+^ T cells, CD19^+^ B cells and CD34^+^ stem cells) in type 1 diabetes risk. Similar efforts going forward, in larger and more diverse populations, should shed light on additional risk loci and variants contributing to the pathogenesis of type 1 diabetes.

Although GWAS and fine mapping efforts have provided much insight into genetic aetiology, the picture remains incomplete. Those type 1 diabetes risk loci, uncovered by such initial classical approaches, remain dominant factors in the genetic picture of disease; however, they do not explain the entire genetic architecture of type 1 diabetes. In order for the power of genetics to fully contribute to risk prediction and discovery of novel therapeutic avenues, additional approaches need to be employed including expansion to multi-ethnic populations, where novel variants have already begun to emerge [9, 10], and into adults, who account for nearly half of those with type 1 diabetes.

## Identifying causal SNPs for type 1 diabetes

A step (of many possible) in determining whether a SNP is a causal variant is to estimate its contribution to gene expression (expression quantitative trait locus, eQTL) [11]. Typical eQTL analysis is equivalent to GWAS but using gene expression as the phenotype. The variant most associated with disease may be near a gene of interest; however, that variant may be regulating the expression of a different, more distal effector gene. This is the situation for a variant in the *FTO* gene that is most strongly associated with obesity which actually regulates *IRX3* [12, 13], and a variant in *TCF7L2* most strongly associated with type 2 diabetes that regulates *ACSL5* [14].

In type 1 diabetes, the 16p13 locus contains strongly associated SNPs spanning introns 10 and 19 of *CLEC16A* [4–6]. A single eQTL was identified in the neighbouring *DEXI* gene, such that the *CLEC16A* SNPs associated with reduced risk of type 1 diabetes correlated with increased *DEXI* expression in monocytes [15]. This result was replicated and identified the most strongly associated variant in *CLEC16A* with expression in B cells, implicating a SNP in *CLEC16A* alters risk of type 1 diabetes through expression of *DEXI* [16]. Differential transcriptome analysis of tolerogenic and mature inflammatory dendritic cells, when overlaid with SNPs associated with type 1 diabetes, identified 11 genes with differential expression [17]; three (*CCR5*, *CTSH* and *RAC2*) with higher expression in tolerogenic dendritic cells compared with mature inflammatory dendritic cells, and eight (*IKZF4*, IKZF1, *SH2B3*, *ORMDL3*, *TYK2*, *IL2RA*, *PTPN2* and *ICOSLG*) with lower expression. These results implicated a role for these disease-associated variants as activators of the immune response in type 1 diabetes.

Although eQTL analysis from peripheral blood provides some insight into possible causal effects of variants associated with type 1 diabetes, immune cell type-specific evaluation (e.g. T-helper 17 cells [Th17], regulatory T cells [Tregs], monocytes) should enhance our understanding of the impact of these variants on target genes. Microarray data from 92 children (25 seroconverters and 67 nonseroconverters) provided longitudinal change in gene expression profiles with development of islet autoimmunity [18]. Gene expression signatures in the first year of life predicted seroconversion with genes that contribute to T cell, B cell and dendritic cell-related immune responses, primarily through a ubiquitin-proteasome pathway. A protein–protein interaction network was linked to type 1 diabetes-associated genes with differentially expressed seroconversion genes, revealing direct interactions with *ERBB3* and *GLIS3*, two type 1 diabetes susceptibility genes.

## Gene regulation from a distance

GWAS have delivered many validated loci associated with novel aetiological pathways. But as mentioned above, these SNP associations do not necessarily implicate the closest gene as causal, even if reasonable hypotheses exist between the SNP location and possible gene function (see the *FTO–IRX3* experience, above). Gene expression can be controlled locally or via long-range interactions over large genomic distances. Indeed, many regulatory elements do not control the nearest genes, but, rather, ones residing tens or hundreds of kilobases away. Barriers to detecting the ‘true’ targets of disease-associated SNPs include the limited, but growing public domain genomic data relevant to individual immune cell types, tissue-specific eQTLs, chromatin conformation capture, and emergent variant-to-gene techniques required to identify causal effector genes. Indeed, the identification of the true gene targets is a crucial precursor to a rational therapeutic and diagnostic development leveraging genetic information.

The majority of type 1 diabetes-associated SNPs map to regions distant from genes [8]; thus, genomic maps are needed that determine how these SNPs might influence chromatin accessibility, transcription factor binding and the physical structure of the genome in order to identify the target genes important in disease. The vast majority (>95%) of the human genome is inaccessible to the machinery that regulates gene expression [19]; thus, essentially all transcription factor and RNA polymerase binding is concentrated at open chromatin regions. Therefore, maps of open, transposase-accessible chromatin (e.g. generated using the assay for transposase-accessible chromatin using sequencing [ATAC-Seq] [20] at the multi- or single-cell level) can identify regions of potential regulatory significance across multiple tissues. One example used the open chromatin landscapes of follicular helper T cells (TFH) from human tonsil to identify functional variants implicated by GWAS of systemic lupus erythematosus (SLE) [21]. The proxies of SLE ‘sentinel’ SNPs (those SNPs in strong linkage disequilibrium with the most associated SNP from the GWAS) are highly enriched in the open chromatin of TFH cells, a cell type critical for the development of autoantibodies characteristic of SLE, compared with naive CD4^+^ T cells. These accessible SLE SNPs were more likely to be located in the promoters of genes highly expressed in TFH cells and involved in other systemic autoimmune disorders, including type 1 diabetes. Genetic variation in a promoter can influence expression of its downstream gene, given proximity of the disease-associated SNPs and recognised cis effects.

**Fig. 1:**
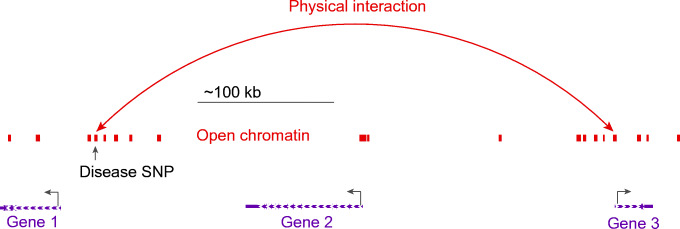
Possible promoter interactions in open chromatin regions, suggesting how SNPs may regulate distant genes through physical contact with non-adjacent promoters, defining likely effector genes and targets. This figure is available as a downloadable slide

A similar prediction, however, is not obvious from maps of open chromatin for more distal SNPs. When disease-associated SNPs are cis eQTLs, they may also physically interact with the promoter (or promoters) that they regulate (for an example, see [22]). These interactions can be detected using chromosome conformation capture, examining not only promoter interactions but also interaction at a distance between promoters, enhancers, silencers and other elements. Chromatin conformation capture-based techniques have the ability to determine whether chromatin ‘looping’ contributes to human disease at key locations associated with complex traits. In particular, one can now leverage recent findings that have revealed topologically associating domains (TADs) [23], that are largely tissue-independent chromatin compartments within which most enhancer–promoter contacts occur. Effectively, TADs may establish the boundaries where interactions can occur for a given genomic location, thereby providing a defined shortlist of candidate genes within a locus, among which at least one is highly likely to be a causal effector gene. Whole genome, promoter-focused Capture C, a version of chromatin conformation capture, relates SNPs in the distal regulatory regions to changes in expression of their target genes [24, 25]. High-resolution spatial epigenomic approaches for common complex traits have been able to physically link strongly associated SNPs with their target genes for traits such as SLE [21] and bone mineral density [26, 27], as well as type 2 diabetes and type 1 diabetes (discussed below). These studies demonstrate that 3D regulatory architectures are a consistent feature of highly expressed, lineage-specific genes involved in specialised functions in disease-relevant cell types (Fig. 1).

## Type 1 diabetes distant regulators may differ from those in type 2 diabetes

While prior genetic analysis directly implicated the immune system in genetic risk of type 1 diabetes with lack of enrichment in islet regulatory regions [8], other biological pathways are likely to be involved. The impact of type 1 diabetes associated SNPs on islets, through the targeting of the autoimmune attack on beta cells, may occur prior to clinical onset (e.g. at the initiation or progression stage); alternatively, the type 1 diabetes-associated variants may act directly at the beta cell level in response to a perturbation (e.g. inflammation).

In the context of its type 2 diabetes counterpart [28], islet accessible chromatin peaks aided the identification of active enhancers and promoters through the use of islet samples and 3D chromatin maps by identifying chromatin loops enriched at such genomic features. Of the >6000 islet active enhancers that mapped to a chromatin loop anchor, half were in a loop to a gene promoter. Many of these enhancers looped to a promoter over long distances (mean 165 kb, with 14% over 500 kb, and >3% over 1 Mb). These distal islet enhancer chromatin loops were correlated with islet-specific gene expression (as assessed by the presence of eQTLs), with the strongest evidence observed for active promoter and enhancer SNPs proximal to genes. Genome-wide enrichment of SNPs was observed in active islet regulatory elements within chromatin loops. SNPs associated with type 2 diabetes and in active islet enhancers had, on average, two candidate target effector genes, including some that were >500 kb from the SNP. In a different study [29], experimental perturbation (glucose stimulation) in human islets was used to identify over 1300 enhancer hubs that had features of regulatory domains controlling genes involved in islet cell function and differentiation. Factoring in islet hub SNPs in a polygenic risk score improved identification of individuals with risk of type 2 diabetes, possibly acting through islet gene regulation and insulin secretion pathways.

The effect of inflammatory cytokine (IFN-γ and IL-1β) exposure on the beta cell as a model of initiation of type 1 diabetes has been investigated using 3D mapping approaches to detect novel targets [30]. After cytokine exposure, ~12,500 sites were identified that became accessible and correlated with H3K27ac activity (acetylation at the 27th lysine residue of the histone H3 protein, representing evidence of an active enhancer). Inducible regulatory elements (IREs) were identified, with two-thirds becoming both chromatin accessible and showing enhancer activity after cytokine treatment (neo-IREs), and the other third, which were already accessible, gaining only enhancer activity after cytokine treatment. The proinflammatory cytokine exposure was hypothesised to induce a beta cell response by induction of new distal regulatory elements and binding of transcription factors involved in the inflammatory response. In islet 3D chromatin structure studies, the promoters of 13 genes exhibited strong induction of expression by cytokine exposure, with their promoters gaining chromatin interactions. Distal genomic regions formed specific DNA looping events with new human islet cytokine responsive enhancer–promoter interactions. In this system, variants associated with type 2 diabetes (not type 1 diabetes) overlapped human islet responsive regulatory elements that were not cytokine responsive; however, human islet IREs (induced by cytokine exposure) were enriched for SNPs associated with type 1 diabetes (not type 2 diabetes). In two known type 1 diabetes loci, risk SNPs (rs78037977 in 1q24.3 and rs193778 in 16q13.13) directly overlapped IREs in islets. An allele of rs78037977 at 1q24.3 (common in individuals of European ancestry but rare in those of other ancestries) disrupts cytokine exposure-specific enhancer activity and interacts with *TNFSF18*, a gene ~300 kb from this SNP but activated in islets upon cytokine exposure. At 16q13.13, rs193778 is common in most ancestries (yet monomorphic in Asian populations) and increases enhancer activity, having strong chromatin contact with the promoter of *DEXI*, a gene ~300 kb distal to the sentinel SNP and previously implicated in type 1 diabetes [15, 16].

## Detection and validation of targets of SNPs

Multiple levels of evidence are necessary to determine which SNPs in a locus are likely to be causal and how these variants regulate target effector genes and their products. Candidate SNPs may influence gene expression in appropriate cell types (e.g. detected by applying RNA-seq in immune cells and beta cells) and on transcription (e.g. detected using ATAC-seq for evidence of transposase-accessible chromatin). These and other types of evidence provide a prioritisation for mapping interactions between promoters and distal regulatory elements, with increasing resolution [31]. As discussed, the target gene may not be the nearest neighbour to the causal SNP; furthermore, SNP-connected putative effector genes may have been implicated in other diseases (for example, see [32]), not only providing additional evidence for causality but also providing new therapeutic options.

CRISPR/Cas9 genome editing can be used to confirm that accessible SNPs in one gene reside in novel, *cis*-regulatory elements for other genes with known roles in function disease risk [21]. In type 2 diabetes, the most strongly associated SNP lies within the *TCF7L2* gene [33], with the rs7903146 T allele in intron 3 widely implicated as the causal variant [34]. Informed by observation of chromatin conformation, in addition to influencing *TCF7L2* expression itself, CRISPR/Cas9-mediated editing of rs7903146 dramatically reduced *ACSL5* gene expression and protein levels [14], thus implicating a putative additional effector gene at this locus. *ACSL5* is three genes away from *TCF7L2* and encodes an enzyme (acyl-CoA synthetase long chain family, member 5) with known roles in mammalian fatty acid metabolism. In addition, the knockout mouse for *ACSL5* has increased insulin sensitivity [35]. A similar approach has been employed for epigenome editing of enhancer–promoter assignments in a cell model for type 2 diabetes [29]. With the increasing number of targets being generated, there is a need to validate such variant-to-gene connections at scale. Emerging techniques, such as massively parallel reporter assays [36] and wholesale CRISPR-based perturbation of implicated enhancers, are growing areas that will meet this need.

## From omics to therapeutic targets

Using multiple lines of evidence (genomics, transcriptomics, DNA methylation, perturbation, gene editing), selection and prioritisation of potential therapeutic targets from validated effector gene lists can proceed using a translational ‘bench-to-bedside’ rationale. Gene products not previously implicated in type 1 diabetes, but currently targeted with therapeutics approved by the US Food and drug administration in autoimmune disease settings, could make excellent drug repurposing candidates. Future drug repurposing candidates would include targets with modalities in overlapping biological pathways. Gene products in need of more potent and/or selective agonists or antagonists could be the targets of future drug development efforts.

## Genetics as predictors of stages of type 1 diabetes

Variants associated with type 1 diabetes in prevalent case−control or affected family studies (primarily of young-onset, Northern European ancestry) may not translate to other ancestries, adults or to the initiation and progress of islet autoimmunity. The T1DGC characterised affected family members for genetic contributions to the presence of islet and other organ-specific autoantibodies [37]. HLA alleles (DRB1*0101 and DRB1*0404) and the *PTPN22* rs2476601 (R620W) locus were associated with autoimmunity, while variants in *IFIH1*, *PTPN22*, *SH2B3*, *BACH2* and *CTLA4* were associated with occurrence of multiple autoantibodies [38]. However, this study was conducted in those with existing disease.

Rather than consider risk in terms of single SNPs, genetic risk scores (GRS) sum the risk alleles for each associated SNP (0, 1 or 2), weighted by the effect of the SNP on the phenotype. The use of the GRS in type 1 diabetes permits an assessment of ‘global’ impact of SNPs as a single value, although the composition of the GRS can vary by the number of SNPs included, the population tested and phenotypic definition. In The Environmental Determinants of Diabetes in the Young (TEDDY) study, a type 1 diabetes GRS (T1D-GRS) in the upper quartile increased the risk of developing multiple autoantibodies by the age of 6 years from 5.8% to 11.0% (compared with 4.1% in the lower T1D-GRS quartile) [39]. The risk of developing type 1 diabetes by age 10 years increased from 3.7% to 7.6% in those with a high T1D-GRS (compared with 2.7% in those without). Children in the highest T1D-GRS quartile had an earlier age of onset of islet autoimmunity, a faster progression from single to multiple autoantibodies, and were more likely to develop type 1 diabetes [40]. A high T1D-GRS also predicts proliferation responses to one or more islet antigens [41]. A T1D-GRS has the potential for use in newborn screening for genetic risk of type 1 diabetes [42], classification of adult-onset disease and progression of islet autoimmunity [43]. Furthermore, a T1D-GRS has the potential power to predict when those with type 2 diabetes may require insulin administration [44].

## From bench to bedside to community

Given the low prevalence of type 1 diabetes in the general population (~4/1000), even a highly sensitive and specific test will likely yield low predictive values. While knowledge of associated risk variants and their function and target effector genes offers the opportunity to identify novel therapeutic pathways [45], there is uncertainty as to how genetics can drive risk prediction. The overall risk of type 1 diabetes is, in part, due to genetic factors, so a high T1D-GRS does not mean one is destined to develop type 1 diabetes per se, just as a low T1D-GRS is not necessarily protective from the disease. Nonetheless, a relatively simple T1D-GRS can identify >10% risk of developing autoimmunity before the age of 6 years [42], making genetic screening a real possibility [46]. Currently, genetics is the only tool detecting those at risk prior to development of islet autoimmunity, until environmental factors (or other novel biomarkers) that trigger the autoimmunity are identified. It is likely that population screening will use a combination of genetics with emergent risk factor testing to determine those eligible for intervention (e.g. intervention trials prior to disease onset, such as with oral insulin therapy) and is being tested now [47]. With the ever-expanding reliance on the merger of electronic health records with biobanks, these research directions could be directly applied to prediction, intervention and treatment in diverse and previously underserved populations.

## Conclusions

The genetic basis of type 1 diabetes is becoming increasingly clear, particularly in Northern European paediatric populations. These gains have yet to impact prediction, prevention and treatment strategies. The vast majority of genetic variants associated with type 1 diabetes reside in regulatory regions of the genome (not in coding regions of genes). Thus, integration of genomics with gene expression, epigenetics and 3D mapping of interactions within the genome are needed to determine the likely target effector genes involved in type 1 diabetes pathogenesis. Identification of new classes of genetic variants associated with type 1 diabetes may enhance the application of genetic risk scores in many ways, from prediction of risk to the need for insulin treatment in type 2 diabetes. Many needs remain, including studies in ethnically diverse populations and in adults, all of which may provide the biological insights needed to translate genomic findings into precision diabetes medicine.

## Electronic supplementary material

Figure slide (PPTX 64.7 kb)

## Abbreviations

eQTL: Expression quantitative trait locus
GRS: Genetic risk score
GWAS: Genome-wide association scan
IRE: Inducible regulatory element
SLE: Systemic lupus erythematosus
T1DGC: Type 1 Diabetes Genetics Consortium
TFH: Follicular helper T cells
WTCCC: Wellcome Trust Case Control Consortium
