# Supplementary material for: Next steps in the identification of gene targets for type 1 diabetes
Source: Diabetologia. 2020 Aug 14;63(11):2260–9. doi: 10.1007/s00125-020-05248-8 (PMC7527360; doi:10.1007/s00125-020-05248-8)
Supplement: Supplementary file 1 — (PPTX 64.7 kb) [file 125_2020_5248_MOESM1_ESM.pptx]

## Slide 1
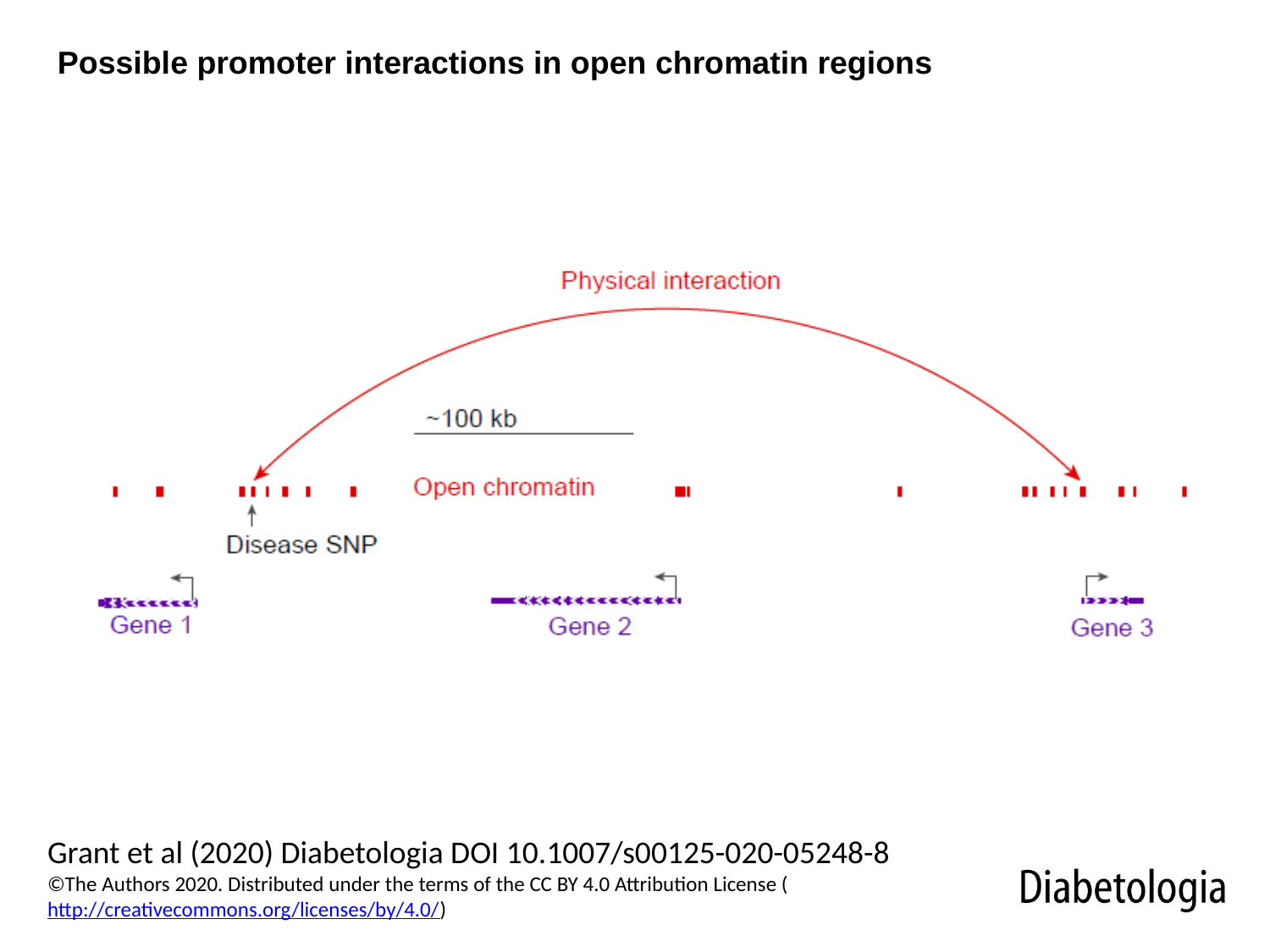

Possible promoter interactions in open chromatin regions
Grant et al (2020) Diabetologia DOI 10.1007/s00125-020-05248-8
©The Authors 2020. Distributed under the terms of the CC BY 4.0 Attribution License (http://creativecommons.org/licenses/by/4.0/)
